# The sensory amphidial structures of *Caenorhabditis elegans* are involved in macrocyclic lactone uptake and anthelmintic resistance

**DOI:** 10.1016/j.ijpara.2018.06.003

**Published:** 2018-11

**Authors:** Antony P. Page

**Affiliations:** Institute of Biodiversity, Animal Health and Comparative Medicine, University of Glasgow, Glasgow G61 1QH United Kingdom

**Keywords:** Ivermectin, Anthelmintic resistance, *Caenorhabditis elegans*, Nematode, Amphids, *Haemonchus contortus*, Intraflagellar transport

## Abstract

Parasitic nematodes represent formidable pathogens of humans, livestock and crop plants. Control of these parasites is almost exclusively dependent on a small group of anthelmintic drugs, the most important of which belong to the macrocyclic lactone class. The extensive use of these drugs to control the ubiquitous trichostrongylid parasites of grazing livestock has resulted in the emergence of both single and multi-drug resistance. The expectation is that this resistance will eventually occur in the human parasites such as the common and debilitating soil transmitted nematodes and vector-borne filarial nematodes. While the modes of action of anthelmintics such as ivermectin, have been elucidated, notably in the model nematode *Caenorhabditis elegans*, the molecular nature of this resistance remains to be fully determined. Here we show that the anterior amphids play a key role in ivermectin uptake and mutations in these sensory structures result in ivermectin resistance in *C. elegans*. Random genetic mutant screens, detailed analysis of existing amphid mutants and lipophilic dye uptake indicate that the non-motile ciliated amphid neurons are a major route of ivermectin ingress; the majority of the mutants characterised in this study are predicted to be involved in intraflagellar transport. In addition to a role in ivermectin resistance, a subset of the amphid mutants are resistant to the non-related benzimidazole class of anthelmintics, raising the potential link to a multi-drug resistance mechanism. The amphid structures are present in all nematodes and are clearly defined in a drug-sensitive strain of *Haemonchus contortus*. It is predicted that amphidial drug uptake and intraflagellar transport may prove to be significant in the development of single and multi-drug resistance in the nematode pathogens of veterinary and human importance.

## Highlights

• The anterior sensory ‘antennae’ of nematodes, the amphids, are involved in ivermectin uptake and transport.
• Genetic screens in *Caenorhabditis elegans* for resistance to ivermectin predominantly affect dye uptake by the amphids.
• Independently derived chemosensory, osmotic avoidance and dye-filling mutants are also resistant to ivermectin.
• A subset of amphid mutants are also resistant to the unrelated anthelmintic, albendazole.
• Many resistance genes are linked to intraflagellar transport and ciliogenesis, and have homologues in *Haemonchus contortus*.

## Graphical abstract

**Figure:**
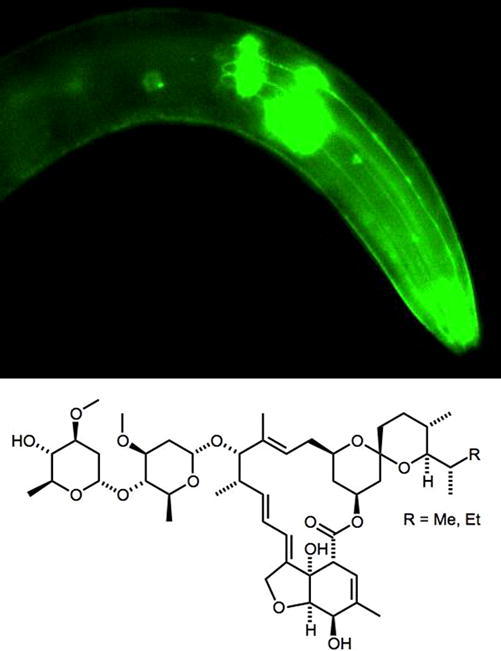

## 1 Introduction

Nematodes represent important pathogens of humans and farmed livestock that have a significant impact on human health and the viability of livestock production. In both human and veterinary medicine, limited control options are available and are heavily dependent on the administration of a small group of broad-spectrum anthelmintic classes of drugs (Geary, 2005) including the macrocyclic lactones. The effectiveness of the macrocyclic lactones such as ivermectin (IVM) in killing nematodes was discovered during the 1970–80s (Campbell and Benz, 1984) and the key role this drug class plays in the control of veterinary, and human, parasitic nematode infections was recognised through the award of the Nobel Prize for Physiology and Medicine in 2015 (https://www.nobelprize.org/prizes/medicine/2015/summary/). Resistance to the broad-spectrum anthelmintics such as IVM, levamisole (LEV) and albendazole (ABZ) is now becoming commonplace in veterinary medicine (Kotze and Prichard, 2016), with real concerns that this situation may ultimately be echoed in human infections (with soil transmitted nematodes, *Onchocerca*, and filarial nematodes) that are currently being controlled through mass drug administration programmes (Geerts and Gryseels, 2000). However, the precise molecular and biochemical mechanisms, particularly in response to the widely used macrocyclic lactone class, remain poorly understood and have been predicted to be multifactorial (Kotze and Prichard, 2016) involving, for example, ligand gated channels, GABA receptors and potentially tubulins. There is also increasing evidence for the involvement of drug transporters and detoxifying enzymes (Kotze and Prichard, 2016). Presently, there are no definitively confirmed IVM resistance genes that have been identified in parasitic nematodes.

The mode of action of IVM was defined by genetic analysis in the model nematode *Caenorhabditis elegans* and found to be via glutamate-gated chloride ion channels, resulting in channel activation and flaccid paralysis (Cully et al., 1994). Genetic studies identified the main glutamate-gated receptor genes, *glc-1*, *avr-14* and *avr-15*, the combined mutation of which resulted in extremely high levels of resistance (Dent et al., 2000). A further six non-glutamate-gated genes were identified in this original IVM resistance screen, four of which (*osm-1*, *osm-5*, *dyf-11* and *che-3*) are related to the sensory neural amphid structures (Dent et al., 2000). The molecular elucidation of resistance mechanisms in field isolates of parasitic nematodes, even following the completion of the genome sequence in species such as *Haemonchus contortus* (Laing et al., 2013, Schwarz et al., 2013), has not, to date, directly linked specific glutamate-gated receptors with IVM resistance.

The *C. elegans* model represents an excellent system in which to examine the role of the amphids in drug uptake and in the generation of resistance. The amphids are a pair of anterior structures comprising non-motile cilia terminating in dendritic process at the nerve ring, are involved in sensing the physical and chemical environment and are analogous in many respects to a vertebrate nose. Defects in these sensilla structures in *C. elegans* are associated with numerous phenotypes including abnormal chemotaxis (Che), abnormal avoidance of high osmolarity (Osm), abnormal dauer formation (Daf), and abnormal dye-filling (Dyf) (Starich et al., 1995). The Dyf mutants are characterised by their inability to take up fluorescent dyes such as FITC and Dil, which in wild-type *C. elegans* are taken up by the anterior amphid and posterior phasmid structures. Laser ablation of these amphidial cells does not affect post-embryonic viability but instead leads to constitutive dauer formation (a long-lived larval stage) (Bargmann and Horvitz, 1991). A previous extensive genetic characterisation of Dyf mutants in *C. elegans* identified 95 new Dyf mutants representing 13 new *dyf* genes but also included 12 new alleles of pre-existing genes, many of which were chemotaxis defective (Starich et al., 1995).

In this present study, forward genetic screens were applied to investigate the link between Dyf mutants and IVM resistance. To support this link, the IVM resistance properties of a selection of previously identified *C. elegans* amphid and chemosensory mutants that were not known to be associated with anthelmintic resistance, were examined and identified as novel IVM resistance genes. The amphidial uptake of fluorescent dyes and the presence of the IVM resistance gene homologues in the parasitic nematode *H. contortus* were also examined.

## 2 Materials and methods

All fine chemicals were purchased from Sigma (UK) unless otherwise stated.

### 2.1 Nematode strains and maintenance

*Caenorhabditis elegans* strains were supplied by the *C. elegans* Genetics Centre, USA and were maintained on Nematode Growth Media (NGM) agar plates supplemented with *Escherichia coli* bacteria OP50-1 following standard techniques http://www.wormbook.org/toc_wormmethods.html. The following strains were used in this study; N2, CB3323 (*che-13*), CB3332 (*che-12*), SP1205 (*dyf-1*), MT3559 (*dyf-9*), CB3687 (*che-14*), MX52 (*bbs-8*), SP1745 (*dyf-5*), PR811 (*osm-6*), PR672 (*che-1*), SP1234 (*dyf-2*), PR813 (*osm-5*), PR802 (*osm-3*), SP1237 (*dyf-4*), CB3330 (*che-11*), DR86 (*daf-19*), CB1126 (*che-6*), SP1678 (*dyf-13*), SP1709 (*dyf-10*), SP1603 (*dyf-3*), SP1735 (*dyf-7*), CB398 (*mec-8*), SP1712 (*dyf-6*), CB1033 (*che-2*), CB1387 (*daf-10*), CB1066 (*mec-1*), SP1713 (*dyf-11*), VC837 (*bbs-1*), CB3329 (*che-10*), MT3645 (*osm-12*), CB1124 (*che-3*), CB1253 (*che-3*), CB4856 (Hawaiian strain) and PR808 (*osm-1*).

The drug-sensitive *H. contortus* strain, MHco3(ISE), was kindly provided by Dave Bartley and Alison Morrison from the Moredun Research Institute, UK. Embryos were purified from faecal samples derived from mono-specifically infected donor lambs via saturated salt flotation. The eggs were hatched to L1s and developed to L2s and L3s by culturing on NGM agar OP50-1 as per *C. elegans*.

### 2.2 IVM, abamectin and ABZ resistance test in *C. elegans*

IVM and abamectin stocks were made up in solvent (DMSO final volume 1%) and added to cooled (50 °C) NGM media, at 8–50 nM final concentrations prior to pouring 5 cm plates then seeding with OP50-1 and plates were used the next day. For resistance assays either 50 purified embryos, five L4s or five young adults were placed on drug selection plates. Resistance was assessed as F1 survival after 3 to 5 days. All wild type (N2) and Hawaiian (CB4856) nematodes were dead on 8–10 nM IVM after 5 days and failed to progress beyond L1s, but all resistance mutants survived these conditions, developing to adults. For ABZ, the same process was followed, except 100 µM (or 150 µM) ABZ was added to the cooled NGM plates, and drug effectiveness was determined as complete paralysis of all N2 worms after 3–5 days whereas resistant worms were active and moved freely on the ABZ plates.

### 2.3 EMS mutagenesis

Four separate chemical mutagenesis screens (Table 1) were carried on wild type N2 *C. elegans* with ethyl methanesulfonate (EMS) following previously published methods (Brenner, 1974). Briefly, synchronised L4s were washed from plates in M9 buffer and incubated in 50 mM EMS for 4 h at 20 °C, washed in M9, and allowed to recover on NGM plates overnight. Twenty adults per plate were transferred to 60 × 9 cm Petri dishes for each screen, overall representing a large mutagenic screen at approximately 400,000 mutagenised F1 genomes. Populations were then allowed to develop to F2 adults which were bleached and resulting eggs were placed on 10 or 50 nM IVM or 50 nM ABZ selection plates. Surviving nematodes from individual plates were maintained as separate mutants.

**Table 1:** *Caenorhabditis elegans* macrocyclic lactone resistance mutants isolated in forward mutagenic screens.

Mutant strain	Resistance screen	Dyf amphid	ABZ R (100 µM)
TP236(ka30)	IVM 10 nM screen 1	Dyf −	No
TP237(ka31)	ABM 50 nM screen 2	Dyf −	No
TP238(ka32)	IVM 10 nM screen 1	Dyf +	No
TP239(ka33)	IVM 10 nM screen 1	Dyf +	No
TP240(ka34)	ABM 50 nM screen 3	Dyf −	No
TP241(ka35)	ABM 50 nM screen 3	Dyf −	Yes
TP242(ka36)	ABM 50 nM screen 3	Dyf −	No
TP243(ka37)	ABM 50 nM screen 3	Dyf −	No
TP249(ka43)	ABM 50 nM screen 3	Dyf −	No
TP250(ka44)	IVM 10 nM screen 1	Dyf −	No
TP271(ka63)	IVM 10 nM screen 4	Dyf −	No
TP272(ka64)	IVM 10 nM screen 4	Dyf −	No
TP273(ka65)	IVM 10 nM screen 4	Dyf −	No
TP274(ka66)	IVM 10 nM screen 4	Dyf −	No
TP275(ka67)	IVM 10 nM screen 4	Dyf −	No
TP173(ka98)	IVM 10 nM screen 1	Dyf −	No
TP153(ka99)	IVM 10 nM screen 1	Dyf −	No

Dyf, amphid dye-filling; IVM, ivermectin; ABM, abamectin; ABZ R, albendazole resistant.

### 2.4 Mutant single nucleotide-polymorphism (SNP) mapping and whole genome sequencing

Two IVM-resistant mutants, TP238*(ka32)* and TP239*(ka33)*, were selected for whole genome sequencing following backcrossing to a polymorphic SNP-rich Hawaiian *C. elegans* strain (CB4856) essentially as described (Doitsidou et al., 2010). CB4856 are as sensitive to 10 nM IVM as wild type N2, as they also fail to develop beyond the L1 stage. Briefly, each hermaphrodite mutant strain was crossed with male CB4856 and F1 progeny were selected following single worm PCR with the following Hawaiian genotyping primers (F1: GGGATCACCATATTTGGTAAGA, R1: CATCGTGATGAAAAGTTGATGAC and F2: CGAGTAATGCTTCAGACAAGT). Multiple (>50) single F2 L4 progeny were then cultured on 10 nM IVM plates and resistant populations were selected after 5 days, then reselected for a further 5 days on a second set of 10 nM IVM plates. The resistant Hawaiian crossed nematodes from 50 individual overgrown plates were then washed off, pooled and genomic DNA was prepared using a Gentra Puregene Tissue kit (Qiagen, UK) and was cleaned and concentrated using a genomic DNA clean and concentrator kit (Zymo, USA). The genomic DNA was sequenced via Illumina technology on the MiSeq system using the MiSeq Reagent Kit v3 (600-cycle). The sequence data was analysed using the CloudMap pipeline (Minevich et al., 2012) and the reads were aligned to the *C. elegans* genome version WS220.64. Analyses were performed on the Galaxy instance of Glasgow Polyomics, University of Glasgow, UK (Giardine et al., 2005).

### 2.5 Dil (1,1′-dioctadecyl-3,3,3′,3′-tetramethylindocarbocyanine perchlorate) staining

All nematodes (*C. elegans* adults and *H. contortus* L2s) were examined for amphidial Dil dye (Thermofisher, UK) uptake. Nematode larvae were purified as in Section 2.1, washed in M9 buffer and incubated in 10 µg/ml of Dil for 3 h at room temperature in the dark prior to washing with M9 and transferring to NGM OP50-1 plates overnight. Nematodes were then picked to 2% agarose pads for microscopy and imaging.

### 2.6 RNA interference (RNAi) and IVM resistance selection

RNA interference (RNAi) clones (F18C12.1 and B0365.7) corresponding, respectively, to the IVM resistance mapping candidates TP238 *che-3* (*ka32*) and TP239 *dhc-3* (*ka33*), were recovered from the *C. elegans* RNAi feeding library (Source Bioscience), streaked on 100 μg/ml of ampicillin and 12.5 μg/ml of tetracycline Lauria Broth and overnight cultures were subsequently prepared in Lauria Broth medium with 100 μg/ml of ampicillin. N2 worms were grown to the adult stage, then bleach treated to recover embryos and 50 embryos were added to each 5 cm NGM plate. The RNAi plates, *che-3* (F18C12.1), *dhc-3* (B0365.7) and empty vector (L4440), all contained 100 μg/ml of ampicillin and 1 mM Isopropyl β-d-1-thiogalactopyranoside (IPTG). Bacterial RNAi clones were added and allowed to grow overnight, and N2 embryos were added to RNAi plates and allowed to develop to adulthood for 3 days. Five adults were then moved to a new RNAi plate (F18C12.1 to F18C12.1, B0365.7 to B0365.7 and L4440 to L4440) supplemented with 10 nM IVM and survival and development was assessed over 3 days.

### 2.7 Imaging and microscopy

All nematodes were either viewed on plates using a Zeiss benchtop microscope fitted with a Canon Sureshot camera or were mounted on 2% agar pads on slides and viewed under Differential Interference Contrast (DIC) or fluorescence optics on a Zeiss Axioscop2 and imaged with a Zeiss AxioCam camera and Axiovision software.

## 3 Results

### 3.1 IVM resistance and Dil dye uptake

**Fig. 1:**
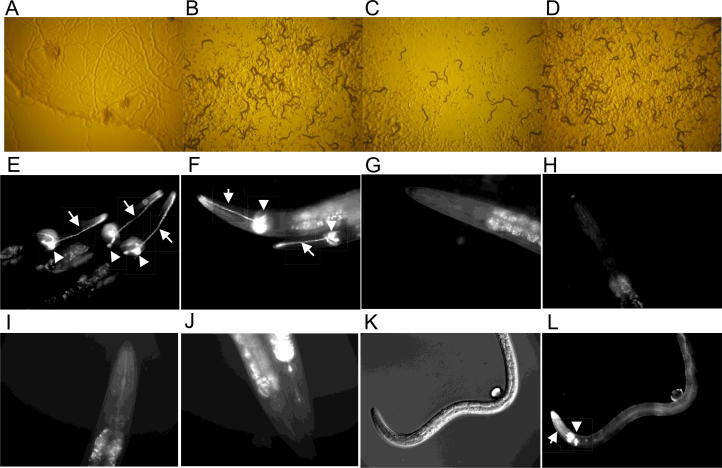
Ivermectin resistance and amphid dye uptake in *Caenorhabditis elegans*. N2 wild type *C. elegans* after 5 days culture on 10 nM IVM, all nematodes are completely dead (A), or 0 nM IVM (1% DMSO), healthy population (B). TP239(*ka33*), resistant population (C) and CB3330 *che-11(e1810)* after 5 days culture on 10 nM IVM, representing strong resistance (D). A–D ×40 magnification. Dil amphid dye uptake in wild type *C. elegans* N2 (E), and ivermectin-resistant mutant TP239(*ka33*) (F), and exclusion in ivermectin-resistant mutant TP249(*ka43*) (G) and chemosensory mutant CB3330 *che-11(e1810)* (H), the osmotic avoidance mutant PR808 *osm-1*(p808) (I) and the dye-filling mutant SP1735 *dyf-7*(m537) (J). Dil is also taken up by the amphids and binds the nerve ring of the L2 stage of *Haemonchus contortus* ivermectin- and benzimadazole-sensitive strain (MHco3 ISE), differential interference contrast image (K), and U.V. image (L). Arrows depict amphids and arrow heads the nerve ring. E–L magnification ×200.

In this study, a robust IVM resistance assay was developed to examine the correlation between low level (10–50 nM) macrocyclic lactone resistance and Dil dye exclusion from the sensory amphids. When wild-type *C. elegans* embryos, L4s or adults were placed on 10–50 nM IVM supplemented plates, no animals survived after 5 days (Fig. 1A). This is in contrast to confluent growth noted on non-drug supplemented plates (with 1% DMSO) where F1 populations of wild-type embryos, active larvae and adults were evident (Fig. 1B). Similar drug selection plates were then used to isolate IVM- and abamectin-resistant mutants following chemical mutagenesis (Fig. 1C) and to test pre-existing amphid defective mutants that had not previously been assessed for IVM resistance (Fig. 1D).

In addition to drug selection, the lipophilic dye Dil was applied to examine the integrity of the amphid sensory structures in live nematodes. In wild type *C. elegans* the fluorescent dye bound to the amphid channels and the nerve ring structures in all larval stages and adults (Fig. 1E), whereas this dye was excluded from the majority of IVM-resistant (Fig. 1G; Table 1) and amphid defective mutants (Fig. 1H–J; Table 2). It is intriguing to note that this dye also binds to the amphid structures and nerve ring of the L2 stage of the *H. contortus* IVM- and benzimidazole-sensitive strain (MHco3 ISE) (Fig. 1L). Randomly picked L2s (39/39) displayed this high level of dye filling following staining with Dil.

**Table 2:** Many *Caenorhabditis elegans* amphid defective mutants are resistant to ivermectin and albendazole. Bold represents strong resistance (characterised as vigorous growth on 10 nM ivermectin, comparable with non-drug plates and underlines represent previously identified dye-filling (Dyf) mutants that are resistant to ivermectin.

Gene (homology)	Strain (allele)	Mutation/effect	IVM R	ABZ R	*H. contortus* homologue (% ID and aa length)
*CHEmosensory mutants*					
**che-1** (zinc finger Trans factor)	PR672(p7672)	Substitution/Nonsense	Yes	Yes	No

che-2 (G-protein WD repeat)	CB1033(e1033)	Unknown	Yes	No	Yes (56% over 281 aa)
che-3 (Dynein HC avr-1, IFT)	CB1124(e1124)	Substitution/Nonsense	Yes	No	Yes (61% over 696 aa)
che-6 (cyclin channel)	CB1126(e1126)	Unknown	No	No	Yes (58% over 238 aa)
che-10 (rootelin, IFT)	CB3329(e1809)	Unknown	Yes	No	No
**che-11** (large unfamiliar, IFT)	CB3330(e1810)	Substitution/Nonsense	Yes	Yes	Yes (68% over 357 aa)

che-12 (creserin, IFT)	CB3332(e1812)	Splice site substitution	Yes	No	Yes (56% over 236 aa)
**che-13** (IFT57/Hippi)	CB3323(e1815)	Substitution/Nonsense	Yes	Yes	Yes (53% over 209 aa)

che-14 (sterol sensing)	CB3687(e1960)	Splice site substitution	Yes	No	Yes (59% over 391 aa)

*OSMotic avoidance mutants*					
osm-1 (WD repeat IFT)	PR808(p808)	Unknown	Yes	No	Yes (55% over 714 aa)
osm-3 (kinesin family IFT)	PR802(p802)	Substitution/Nonsense	Yes	No	Yes (87% over 328 aa)
**osm-5** (polaris IFT)	PR813(p813)	Substitution/Nonsense	Yes	Yes^a^	Yes (69% over 391 aa)
osm-6 (novel IFT)	PR811(p811)	Splice site substitution	Yes	No	Yes (52% over 233 aa)
osm-12 (bbs7 IFT)	MT3645(n1606)	Substitution/Nonsense	Yes	No	Yes (59% over 270 aa)

*DAuer DeFectice mutants*					
**daf-10** (WD repeat IFT)	CB1387(e1387	Substitution/Nonsense	Yes	Yes^a^	Yes (60% over 313 aa)
daf-19 (RFX transfer factor)	DR86(m86)	Substitution/Nonsense	Yes	No	Yes (69% over 186 aa)

*DYeFilling defective mutants*					
dyf-1 (novel IFT)	SP1205(mn335)	Insertion/ frameshift	Yes	No	Yes (60% over 353 aa)
dyf-2 (WRD19 IFT)	SP1234(m160)	Substitution/Nonsense	Yes	No	Yes (58% over 250 aa)
**dyf-3** (CLUAP protein IFT)	SP1603(m185)	Substitution/Nonsense	Yes	Yes	Yes (58% over 231 aa)
**dyf-4** (uncloned)	SP1237(m158)	Unknown	Yes	Yes^a^	uncloned
dyf-5 (map kinase)	SP1745(mn400)	Substitution/Nonsense	Yes	No	Yes (85% over 315 aa)
dyf-6 (novel IFT)	SP1712(m175)	Substitution/Nonsense	Yes	No	No
**dyf-7** (ZP protein)	SP1735(m537)	Unknown	Yes	Yes^a^	Yes (66% over 132 aa)
dyf-9 (uncloned)	MT3559(n1513)	Unknown	Yes	No	uncloned
dyf-10 (uncloned)	SP1709(e1383)	Unknown	Yes	No	uncloned
dyf-11 (IFT54 homolog IFT)	SP1713(mn392)	Substitution/Nonsense	Yes	No	No
dyf-13 (novel IFT)	SP1678(mn396)	Splice site substitution	Yes	No	Yes (46% over 291 aa)

*MEChanosensory defective*					
mec-1 (EGF/Kunitz)	CB1066(e1066)	Splice site substitution	Yes	No	Yes (57% over 227 aa)
**mec-8** (RRM domain)	CB398(e398)	Substitution/Nonsense	Yes	Yes	Yes (85% over 95 aa)

*Bardet-Biedl syndrome protein*					
**bbs-1** (BBS1 ortholog IFT)	VC837(ok1111)	Deletion coding	Yes	Yes^a^	Yes (41% over 168 aa)

bbs-8 (TPR protein IFT)	MX52(nx77)	Deletion coding	Yes	No	Yes (58% over 356 aa)

*Novel EMS derived IVM resistance gene*					
dhc-3 (Dynein, IFT)	TP239	Insertion/frameshift	Yes	No	No

IVM R, 10 nM ivermectin-resistant; ABZ R, 100 µM albendazole resistant, (^a^, represents ABZ resistance to 150 µM); IFT, intraflagellar transport component homology. *Haemonchus contortus* homologues were investigated by BLASTP using Wormbaseparasite (WBPS10, WS236) with hits shown as % identity over a specified amino acid (aa) length.

### 3.2 IVM resistance forward genetic screens

The four EMS mutagenesis IVM and abamectin resistance screens generated 17 independent mutants that could survive normally lethal concentrations of IVM and abamectin (Table 1). These mutants were all tested for Dil dye uptake, and 15 were found to completely exclude Dil from the amphids and nerve ring (Fig. 1G; Table 1). The two remaining strains, TP238*(ka32)* and TP239*(ka33)* (Fig. 1F), displayed positive but variable Dil uptake, independent of culturing on IVM or non-IVM selection plates. Therefore 15/17 of the new macrocyclic lactone-resistant mutants displayed impaired Dil uptake phenotypes with the remaining two showing variable or near normal Dil uptake (Table 1). Resistance to both macrocyclic lactones, abamectin and IVM, was found to be linked to defective amphids in this nematode.

### 3.3 Hawaiian mapping and whole genome sequencing of IVM-resistant, dyf-variable mutants

The two strains, TP238*(ka32)* and TP239*(ka33)*, were selected for whole genome sequencing as they displayed variable Dil uptake and differed from the remaining 15, Dil excluding, macrocyclic lactone resistance mutants. This analysis involved a Hawaiian, SNP-rich strain (CB4856) backcrossing strategy, reselection for IVM resistance, pooling of 50 individual backcrossed and resistant strains, DNA purification and whole genome sequencing. This powerful approach followed published methods (Doitsidou et al., 2010) and allowed rapid mapping, resulting in the identification of a novel *che-3* allele (TP238, *ka32*) and a novel IVM resistance gene *dhc-3* (TP239, *ka33*). The whole genome sequencing and mapping data is presented Supplementary Files S1 and S2. TP238(*ka32*) encodes a C/T transition at position 8,070,133 on Chromosome I, that results in a G/R change in codon 2568 of exon 30 of this dynein heavy chain encoding gene. The mutation in *dhc-3* of TP239(*ka32*), also a dynein heavy chain encoding gene, was the result of a 104 bp Chromosome V deletion in the coding sequence of B0365.7 (13,150,172 to 13,150,276) which disrupts the final eight exons of this very large transcript. Both of these mutations are predicted to affect intraflagellar transport and are expected to be associated with amphid structure in spite of the fact that Dil dye amphid uptake was variable in these mutants.

**Fig. 2:**
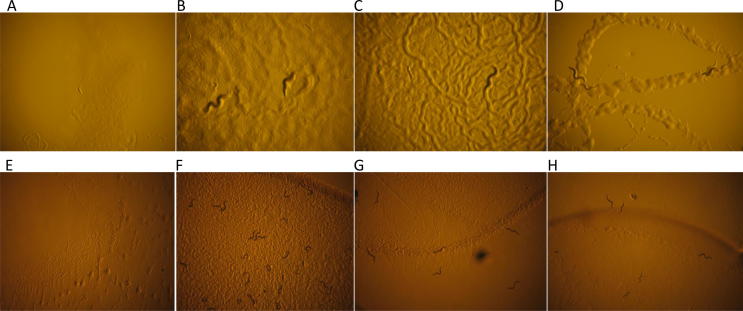
Confirmation of ivermectin resistance genes identified by whole genome sequencing by testing on 10 nM ivermectin plates. Wild type N2 were killed and failed to progress beyond L1 (A), compared with TP238 which survive to adult stage on 10 nM IVM (B). TP238(*ka32*) mapping candidate alleles CB1124(*che-3*, *e1124*) (C) CB1253 (*che-3*, *e1253*) (D) also survive to adult stage on 10 nM IVM plates. A–D, 20 embryos added per plate and left or 3 days, (magnification ×80). RNA interference (RNAi) testing of candidate genes for resistance to 10 nM ivermectin. E–H (×40). Empty vector RNAi control (L4440) treated N2 killed on 10 nM ivermectin (E) compared with resistant strain TP239(*ka33*) (F) which progresses to adulthood. TP238(*ka32*) mapping candidate *che-3* (F18C12.1 RNAi) fed to N2 strain (G) and TP239(*ka33*) mapping candidate *dhc-3* (B0365.7 RNAi) fed to N2 strain (H); both survived to adulthood on 10 nM ivermectin.

The mapping candidates were examined further by obtaining two of the available mutant alleles of *che-3* and by applying an RNAi approach to determine if knockdown of both candidate genes would confer IVM resistance in the aforementioned IVM plate assays (Fig. 2). Pre-existing mutants were only available for *che-3* but not *dhc-3* (www.wormbase.org), and two independent *che-3* alleles (*e1124* and *e1253*) were tested and found to survive to adulthood on 10 nM IVM plates (Fig. 2C and D), being comparable to the TP238(*ka32*) mutant strain (Fig. 2B) and in marked contrast to the wild type strain N2, which arrests at L1 under identical selection conditions (Fig. 2A). The mapping data was also supported for both *che-3* and *dhc-3* following and RNAi approach whereby resistance to 10 nM IVM was conferred to the normally susceptible N2 strain, by pre-exposing this strain to corresponding RNAi feeding constructs prior to exposure to 10 nM IVM. The RNAi empty feeding vector control did not permit survival on 10 nM IVM (Fig. 2E) in contrast to the TP238(*ka32*) (Fig. 2B) and TP239(*ka33*) (Fig. 2F) mutant strains which survived this treatment. Similarly, the TP238(*ka32*) candidate *che-3*(F18c12.1) (Fig. 2G) and the TP239(*ka33*) candidate *dhc-3*(B0365.7) RNAi feeding (Fig. 2H) of the N2 strain both permitted survival and allowed development to adulthood on the normally lethal 10 nM IVM plates.

### 3.4 IVM resistance screen based on pre-existing amphid defective mutants

The strong link between amphid mutants and IVM resistance was further investigated by examining the ability of previously identified amphid mutants, that were not previously reported to be resistant to IVM, to survive on 10 nM IVM plates (Table 2). A subset of these mutants (*che-1*, *che-11*, *che-13*, *osm-5*, *daf-10*, *dyf-3*, *dyf-4*, *dyf-7*, *mec-8* and *bbs-1*) are described as having strong resistance on 10 nM IVM plates, as they exhibited comparable growth rates to those cultured on non-drug plates. An example of a novel, strong IVM-resistant chemosensory defective strain *che-11(e1810)* (Fig. 1D) and its ability to exclude Dil from the amphids (Fig. 1H) is presented. This linkage of IVM resistance to Dil exclusion is also shown for a representative osmotic avoidance mutant *osm-1(p808)* (Fig. 1I), and a dye-filling mutant *dyf-7(m537)* (Fig. 2J), both of which are resistant to IVM (Table 2). In total, 31 mutant strains from six different gene classes were tested and only one, *che-6(e1126)*, was found to be sensitive to 10 nM IVM. All 31 genes have been described as being dye-filling defective (Starich et al., 1995), but only four (*che-3*, *osm-1*, *osm-5* and *dyf-11*) had previously been described as being resistant to IVM. Therefore, this study has identified 26 new IVM resistance genes from *C. elegans*, the majority of which are substitutions or nonsense mutations in genes that encode proteins highlighted by homology to be involved in intraflagellar transport within the amphids. In total, from these investigations there are 20 genes that are listed as encoding proteins involved in intraflagellar transport that when mutated result in IVM resistance (Table 2).

It is significant to note that homologues for 24 of the 29 molecularly defined mutants are found in the almost completely sequenced genome of the parasitic nematode *H. contortus* (Table 2). This analysis was done by BLASTP analysis of the *C. elegans* homologues against the Wormbase parasite database (WBPS 10). The absence of *che-1*, *che-10*, *dyf-6*, *dyf-11* and *dhc-3* may reflect the fact that the currently available genomes of *H. contortus* are incomplete (Laing et al., 2013, Schwarz et al., 2013). These homologues include 7/9 chemosensory mutants, 5/5 osmotic avoidance mutants, 2/2 dauer defective mutants, 6/8 dye-filling mutants, 2/2 mechanosensory mutants and 2/2 Bardet-Biedl syndrome mutants (Table 2).

### 3.5 ABZ resistance screen

In the field of veterinary parasitology there are many examples of common trichostrongylid parasites, closely related to *C. elegans*, that are resistant to combinations of different classes of anthelminthic drugs including the macrocyclic lactones and the benzimidazoles (Sargison et al., 2007). To address multi-drug resistance in the amphid mutants of *C. elegans*, nematodes were cultured on plates supplemented with 100 µM ABZ and populations were assessed for paralysis (immotile) and death after 3–5 days of exposure. Nematodes from the mutagenesis screen (Table 1) and the defective amphid mutant collection, (Table 2) were assessed for resistance to ABZ. From the forward genetic screen only one strain, TP241(*ka35*), was resistant to both the macrocyclic lactones and the benzimidazole drug ABZ (Table 1). From the amphid mutant collection, 10 of the 31 mutants were also found to be resistant to 100 µM ABZ (Table 2), with at least one member of each gene class having this multiple resistance, and five of these (*che-11*, *osm-5*, *daf-10*, *dyf-3* and *bbs-1*) also being predicted by homology to play roles in intraflagellar transport. Five of the amphid-related mutants (*osm-5*, *daf-10*, *dyf-4*, *dyf-7* and *bbs-1*) exhibited high levels of resistance, surviving and remaining motile on 150 µM ABZ plates. These five mutants were also denoted as having strong resistance to IVM (Table 2). Of the 10 shared IVM- and ABZ-resistant mutants, nine have been molecularly defined and eight of these genes have homologues present in the *H. contortus* genome, five of the eight being predicted to be associated with intraflagellar transport (Table 2).

## 4 Discussion

This study clearly indicates that the amphids represent the main route of IVM uptake into *C. elegans*, and that mutation of genes that cause amphid defects, predominantly in the intraflagellar transport machinery, are the primary mode of IVM resistance in this nematode species. This genetic study strongly supports this hypothesis as four random forward genetic screens, comprising >400,000 mutagenised genomes, generated amphid defects associated with resistance to the macrocyclic lactones. All the drug-resistant mutants from these screens had defects related to the amphids. These mutants were selected for resistance on 10 nM and 50 nM IVM and 50 nM abamectin, a concentration that is comparable to the clinical bloodstream concentration required to kill ruminant nematodes in the host (Alvinerie et al., 2008). In total, 17 independent drug resistance alleles were isolated and then tested in a dye uptake assay. Fifteen mutants completely excluded the dye, indicating a clear defect in their amphids. The remaining two mutants, which showed a near wild-type phenotype of dye uptake, were analysed by SNP-based mapping and whole genome sequencing methods (Doitsidou et al., 2010). This study uncovered mutations in amphid-associated intraflagellar transport genes present within the mapped regions, namely a novel allele of the gene *che-3*, also known as avermectin resistant gene 1 *(avr-1)*, a dynein-heavy chain encoding gene that was isolated in previous avermectin resistance screens (Dent et al., 2000). In addition, a novel IVM resistance associated dynein motor protein component (*dhc-3*, B0365.7), that is a paralogue of *che-3*, was identified in this current screen. This suggests that, although able to take up dyes, the amphids in these two mutants are also likely to be structurally defective or unable to transport the drug effectively. The identity of these mutants has been further confirmed by examining the ability of additional mutant alleles of *che-3* and RNAi knockdown of both *che-3* and *dhc-3* to survive normally lethal concentrations of IVM.

In these unbiased, random genetic screens the 100% (17/17) correlation with amphid defects is remarkable, supporting the hypothesis that defective amphid function causes resistance to clinically relevant concentrations of IVM in this nematode species. It is highly likely that the remaining unmapped and unsequenced mutants will also encode previously identified targets in the amphid structural and intraflagellar transport machinery pathways. It is interesting to note that *che-3* is a very large gene, (25.5 kb coding sequence) and therefore likely to be targeted several times during an EMS screen. Indeed, 21 mutant alleles of *che-3* were isolated during a separate unrelated genetic screen for Dyf mutants (Starich et al., 1995). CHE-3 has been shown to be expressed in the amphids, be involved in intraflagellar transport and through mutant analysis has been shown to be involved in maintaining the structural integrity of the ciliated sensory ending in *C. elegans* (Wicks et al., 2000). DHC-3 is a less well characterised intraflagellar transport component but has been shown to be expressed in the sensory amphids and is predicted to be involved in transport in a defined subset of the amphidial neurones (OLQ) (Hao et al., 2011). Sensory cilia are assembled by intraflagellar transport via anterograde movement of intraflagellar transport particles, whereas retrograde transport is responsible for recycling the transport components back to the base of the cilium and is mediated by the large multi-subunit complexes called dyneins. This transport process and its key components have been elucidated in *C. elegans* and are reviewed in detail elsewhere (Inglis et al., 2007).

To further test the striking association between amphid defects and IVM resistance, available *C. elegans* mutants that had been previously selected based solely on their amphid-defective phenotypes such as Osm, Daf, Che (Starich et al., 1995), and not by resistance to IVM, were also tested for anthelmintic resistance. Thirty-one amphid defective mutant genes comprising members of the Che, Osm, Dyf, Daf, mechanosensation (Mec) and Bardet-Biedl syndrome (Bbs) gene classes were tested for survival on normally lethal 10 nM IVM plates. Mutants of the four genes *osm-1*, *osm-5*, *dyf-11* and *che-3* identified previously as causing IVM resistance (Dent et al., 2000) were included as positive controls. Strains tested were found (30/31) to be resistant to IVM (Table 2). Based solely on the defective amphid phenotype, 26 novel IVM resistance genes have now been identified, unequivocally demonstrating the importance of amphid function for IVM resistance in *C. elegans*. The 26 novel resistance strains include molecularly defined loci, 19 of which are intriguingly listed as genes involved in ciliogenesis and intraflagellar transport (Inglis et al., 2007). Where possible, the reference alleles were selected for testing, with multiple additional amphid mutant alleles being listed as available but so far untested with respect to IVM resistance (www.wormbase.org). Of the alleles tested, the molecular nature of 23 are known, and 15 are nonsense mutations and are therefore predicted to be null alleles; an additional two were splice site mutants and three contained either deletions or insertions with the remaining seven currently listed as uncloned. Intriguingly, 13/30 of the IVM-resistant amphid mutants were also established as being resistant to high concentrations (100–150 µM) of ABZ, a chemically unrelated benzimidazole anthelmintic (Table 1). There are well established mechanisms of nematode resistance to benzimidazoles linking mutations in the drug target, β-tubulin (Kwa et al., 1994); however recent analysis has also identified a wide range of natural mutations in *Caenorhabditis* spp. that are distinct from these known target resistance genes (Zamanian et al., 2018). This observation raises the possibility that common or closely related uptake or transport mechanisms may also be responsible for resistance to diverse anthelmintic compounds and may help to explain certain aspects of the multi-drug resistance observed in field isolates of nematodes of veterinary importance. Indeed, a common theme noted in this cross-resistance was that genes with functional roles in amphid ciliogenesis and intraflagellar transport predominated (eg, *che-13*, *osm-5*, and *bbs-1*; see Table 2).

It can therefore be concluded that the anterior amphidial sensory organs represent a major route of macrocyclic lactone drug uptake and that mutations in this uptake and transport mechanism represent the primary source of drug resistance in this model nematode, and by inference may be involved in the widespread resistance now noted in the parasitic nematodes. In support of this, a correlation has previously been made between the length and organisation of the amphid sensilla and IVM tolerance/resistance in both *C. elegans* (Dent et al., 2000) and the parasitic nematode *H. contortus* (Freeman et al., 2003). Thus shortening, regeneration and/or loss of structural integrity of amphids in nematodes are thought to reduce their ability to transport compounds. Similarly, uptake and toxicity of low levels of aldicarb are associated with the amphids of plant parasitic nematodes (Winter et al., 2002). Previous electron microscopic analysis of *H. contortus* revealed shorter amphid cilia in IVM-resistant strains than in IVM-sensitive nematodes, giving a strong indication that the amphids do indeed play a role in both IVM entry and drug sensitivity (Freeman et al., 2003). A recent study also indicated the importance of the *dyf-7* gene, required for amphid cilia formation, in IVM resistance in *C. elegans* and in several *H. contortus* IVM-resistant strains (Urdaneta-Marquez et al., 2014). These authors also found a link between dye exclusion and IVM resistance in strains of *H. contortus* (Urdaneta-Marquez et al., 2014). A link between the amphids and drug resistance in *C. elegans* was further established recently when long-term exposure to the macrocyclic lactone moxidectin was also found to result in amphid defects (Menez et al., 2016). Other than these reports, the role of the amphids in drug resistance in parasitic nematodes remains a relatively unexplored, but potentially profitable, avenue of future research. It is significant to note that in this study, the lipophilic dye Dil was able to enter and stain the amphidial and nerve ring structures of a known macrocyclic lactone and benzimidazole-sensitive strain of *H. contortus* (MHco3 ISE) (Fig. 1L). It is also significant to note that the majority (83%) of the novel IVM resistance genes identified in this study have a homologue in this important parasite species (Table 1).

The fact that numerous individual amphid associated genes can be mutated to give resistance indicates a large degree of redundancy and may explain why the identification and confirmation of a single gene mutant associated with IVM resistance in the trichostrongylid nematode parasites has, to date, remained elusive. Critical questions arising from these studies are: (i) how widespread are the amphid defects in IVM-resistant field and laboratory isolates of key parasitic nematodes and (ii) how does this uptake mechanism relate to macrocyclic lactone, benzimidazole, levamisole and multi-drug resistance? (iii) What are the consequences of amphid gene mutation on parasite nematodes in the field; would they confer a developmental advantage or disadvantage? (iv) Can this uptake route and mode of transport be exploited in future drug development strategies and finally, (v) can the amphid defective phenotype be utilised as a potential marker of selective or indeed multi-drug resistance in the field?
